# The effect of pupil size on the measurement of corneal birefringence properties: preliminary study

**DOI:** 10.1038/s41598-023-44706-2

**Published:** 2023-10-14

**Authors:** Marcelina Sobczak, Magdalena Asejczyk, Maciej Wilczyński

**Affiliations:** 1 Department of Optics and Photonics, Wrocław University of Science and Technology, Wybrzeże Stanisława Wyspiańskiego 27, 50-370 Wrocław, Poland; 2 School of Optometry, Indiana University, 800 Atwater, Bloomington, IN 47405 United States; 3 Faculty of Pure and Applied Mathematics, Wrocław University of Science and Technology, Wybrzeże Stanisława Wyspiańskiego 27, 50-370 Wrocław, Poland

**Keywords:** Optics and photonics, Applied optics, Predictive markers

## Abstract

We used a partial Mueller matrix polarimeter to measure the corneal anisotropic properties at three pupil sizes (dilated, natural, and constricted). The geometrical parameters of first order isochromes were described by quadrilaterals. These parameters are statistically significantly different between the three pupil sizes. The pupillary size changes do not influence the azimuth angle distribution *α*. The retardation *R* and birefringence distributions show asymmetry in the nasal–temporal cross-section. There are differences between pupil sizes for both nasal and temporal parts of the cornea for these distributions. Iridial light scattering and diffraction might be the reason for these differences.

## Introduction

The stroma is the layer with the most robust anisotropic properties in the cornea. It mainly consists of hundreds of lamellae. The resultant lamellar orientation in the stromal center is random, but towards the limbus, this orientation becomes more ordered, and a preferential axis appears. This creates stronger birefringent properties of this structure^1^. Each lamella contains collagen fibrils embedded in ground substance, each with different refractive indices. This unique stromal organization causes the cornea to become a birefringence medium. Changes in lamellar location hence changes in anisotropic distribution, may indicate lesions or corneal structural diseases, such as keratoconus, that lead to reduced visual acuity or even the need for corneal transplantation. Early detection of these changes with non-invasive methods may help detect and treat their development.

The issue of lamellae location and corneal birefringence concerned many researchers. For instance Rollet in his work suggested that the shape of the interference fringes may reflect the radial character of lamellar orientation in the human cornea (mentioned in Stanworth et al.^2^). His assumption did not find anatomical confirmation. Over time, several models of lamellar orientations were proposed^3–10^. Boote et al. proposed the lamellar distribution model^11^. They showed a significant difference in lamellae distributions in the nasal and the temporal part of the cornea. In their model, the central fibrils are orthogonal to each other and change direction in the peripheral cornea to merge with the tangential fibrils of the highly reinforced limbal annulus. The first mention of corneal birefringence came from Brewster in 1815^12^. He said that human corneas depolarize in all positions. Stanworth and Naylor^13^ using polarimetry technique stated that the cornea can be described as curved uniaxial crystal plate with its optic axis perpendicular to its surface. Most researchers describe corneal polarization properties ditto, which is confirmed via various experiments^14–24^. Naylor^25,26^, Bour et al.^27,28^, using their measurements, concluded that the birefringence in the corneal center is the lowest and that it increases towards the limbus. Van Blokland et al. suggested that in the central area, the cornea behaves as a biaxial crystal with a fast axis perpendicular to its surface and a slow axis located in nasal-lower directions based on their Mueller-matrix ellipsometry^24^. In 2008, Knighton confirmed this hypothesis using Purkinje’s images^29^. Bone et al.^30^ and Misson^1^ using polarizing microscopy in in vitro measurements, stated that cornea might be described as a biaxial medium with low optical retardance. Fanjul-Velez et al. using PS-OCT during in vivo and in vitro studies concluded that the cornea in the center can be described as a linear birefringent biaxial crystal, whereas in the periphery, there is an almost circularly symmetric high-birefringence area^20,21^. In 2016 Westphal et al. suggested that considering the cornea as a linear birefringent medium is insufficient because it does not consider optical rotation. This means that human corneas should be characterized as an elliptically birefringent medium^31^. They also stated that the effect of the depolarization is not significant, especially measured with light shorter than 500 nm. Bueno and Jaroński^17^ also reported that dichroism properties and effects of depolarization are negligible. They mentioned Pelz, who calculated the contribution of the central cornea to the polarization properties using the light reflected from the first lens surface measured in vivo for his PhD thesis. He found that corneal depolarization is not significant, contrary to depolarization due to retina.

The human iris incorporates two layers: the front—fibrovascular layer (anterior limiting layer, stroma of iris, iris sphincter muscle, iris dilator muscle) and the back—pigmented epithelial cells (anterior pigment epithelium, and posterior pigment epithelium). In the first layer are located smooth muscle fibers that enable the dilatation of the pupil (dilator muscle) and its constriction (sphincter muscle). The back surface consists of the heavily pigmented epithelial layer two cells thick. The high content of the pigment blocks light from entering the eye other than through the pupil. We can isolate two main zones in the pupillary plane: the pupillary zone and the ciliary zone. The pupillary zone is the inner region for which the pupillary frill forms the outer boundary of the pupil. The ciliary zone is the remaining part of the iris. The collarette (the thickest iris region) is located at the boundary of the iris’s main zones. It is typically defined as the region where the two iris muscles (the dilator and sphincter) overlap. The structure of the iris is complex. It consists of, i.e., the crypts of Fuchs, which are a series of openings that allow the iris to be bathed in the aqueous humor; the folds that arise during the dilation of the pupil, as well as the radial and structural folds of Schwalbe. The bands of connective tissue generally extend in the radial direction and are called radial furrows. They are responsible for the pattern of fibrovascular tissue in the iris^32^.

The light propagation and absorption within the iridial tissue play an essential role in birefringent measurements using the polarimetry methods, which are based on the assumption that when using these methods, the iris behaves as a reflective surface. Prediction of the exact light behavior in contact with the iris is highly demanding due to the uniqueness of its textural patterns (the probability of two irises agreeing is about one in seven billion^33^). When the light falls on the iridial tissue, we can observe reflection and absorption phenomena on its surface. On account of the iridial structure, light scattering also occurs. Scattering appears in a Rayleigh fashion^34^ where light intensity is proportional to the fourth power of the light frequency^35^. Through the iridial layers, the light is scattered multiple times, and its spatial distribution becomes diffuse. The scattered light distributions carry a near-Lambertian profile^16,36^. Variation in absorption led to the observation of the scattering phenomenon as less perceptible in dark eyes than in those with lighter pigmentation^37^.

Partial Muller matrix polarimetry is a technique of birefringence imaging described in Sobczak et al.^38,39^. It uses a double-pass polarimeter designed to determine selected elements of the Muller matrix at the wavelength of 620 nm. The components of this matrix allow the determination of all birefringent parameters of the anisotropic medium. Its crucial element is the polarization state generator which allows the creation and analysis of different light polarization states. Using the partial Mueller matrix formalism for analyzing received light intensity maps, it is possible to calculate distributions of azimuth angle α and phase difference *γ* (retardation *R*).

The study aimed to investigate whether the topography of the iris as a scattering/reflective element effects the measurement of birefringent properties of the cornea (in the paracentral and peripheral parts) in the double-pass polariscope. This is a compound issue due to the measurements being influenced by several factors e.g. light source location, its angular emission spectrum, and the complexity of the texture pattern of the iris, unique for each eye. Pharmacologically induced changes in iris topography were represented by three sizes of pupil diameter. This preliminary in vivo study aimed to determine the nature of changes in the registered birefringence distributions for narrow, natural, and dilated pupils of healthy volunteers.

## Results

We performed both geometrical and optical analysis of the first order isochrome to check whether the pupil size and iridial surface significantly influence the birefringent measurements. Pupil size changed on average from 12% (for the constricted pupil) through 37% (for natural size pupil) to 68% (for the dilated pupil) of the corneal diameter.

### Geometrical Analysis

**Figure 1:**
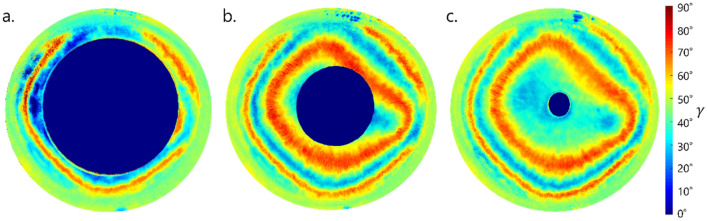
Maps of the exemplary phase difference γ distribution of sample eye for three pupil size: constricted (**a**), natural-sized (**b**) and dilated (**c**).

Figure 1 presents phase difference *γ* distribution maps for an sample eye when the pupil is pharmacologically dilated (Fig. 1a), constricted (Fig. 1c), and when the pupil has its natural size (Fig. 1b). In the most dilated image of the pupil (Fig. 1a), the first order isochrome is barely visible in most cases.

**Figure 2:**
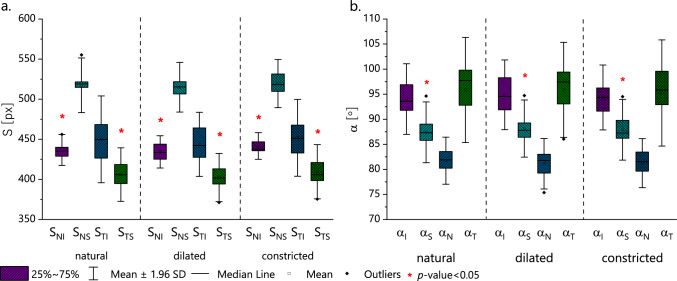
Box-plots of sides lengths (S_TS_, S_TI_, S_NS_, S_NI_) (a) and angles sizes between them (α_T_, α_N_, α_S_, α_I_) (b) for three pupil sizes: constricted, natural-sized and dilated.

Table 1 shows the mean values from all subjects data (± SD) of the side lengths (S_TS_, S_TI_, S_NS_, S_NI_) and angles values (α_T_, α_N_, α_S_, α_I_) of the phase difference distribution maps for the three pupil sizes and the statistical difference between them. For all pupil sizes, the longest side was located in the nasal superior part of the cornea (S_NS_), while the shortest side was in the temporal superior part of the cornea (S_TS_). In the case of the angles of these figures, the angle in the temporal part of the cornea (α_T_) proved to be the most obtuse, and the most acute angle is the one in the nasal part (α_N_) (Fig. 2).

**Table 1:** Mean values (± SD) of the sides lengths (S_TS_, S_TI_, S_NS_, S_NI_) and angles sizes (α_T_, α_N_, α_S_, α_I_) for three pupil size: constricted, natural-sized and dilated and ANOVA (Friedman rang test) results for these parameters.

	Constricted	Natural	Dilated	*p*-value
	Mean ± SD (range)	Mean ± SD (range)	Mean ± SD (range)	
S_TS_ [px]	409 ± 17 (375 ÷ 439)	406 ± 17 (375 ÷ 434)	402 ± 15 (381 ÷ 430)	**0.028**
S_TI_ [px]	452 ± 24 (414 ÷ 494)	450 ± 28 (411 ÷ 494)	444 ± 20 (415 ÷ 469)	0.050
S_NS_ [px]	519 ± 15 (490 ÷ 543)	517 ± 17 (487 ÷ 555)	515 ± 16 (485 ÷ 543)	0.264
S_NI_ [px]	442 ± 8 (430 ÷ 457)	437 ± 10 (426 ÷ 456)	434 ± 10 (419 ÷ 449)	**0.013**
α_T_ [°]	95.2 ± 5.4 (85.1 ÷ 102.2)	95.9 ± 5.3 (85.7 ÷ 101.8)	95.9 ± 4.8 (86.1 ÷ 101.4)	0.472
α_N_ [°]	81.2 ± 2.5 (76.4 ÷ 84.4)	81.7 ± 2.4 (77.3 ÷ 85.0)	81.1 ± 2.6 (75.4 ÷ 84.0)	0.338
α_S_ [°]	87.9 ± 3.1 (82.6 ÷ 94.5)	87.4 ± 3.1 (82.9 ÷ 94.6)	88.1 ± 2.9 (83.6 ÷ 94.7)	**0.017**
α_I_ [°]	94.3 ± 3.3 (89.3 ÷ 99.9)	94.0 ± 3.6 (87.0 ÷ 99.8)	94.9 ± 3.5 (89.1 ÷ 100.7)	0.174

Bold denotes statistical significance.

Using the Friedman Rang test (ANOVA), the lengths of the sides (S_TS_, S_TI_, S_NS_, S_NI_) and angles values (α_T_, α_N_, α_S_, α_I_) were compared between the three pupil sizes. It was shown that the lengths of the sides in nasal inferior S_NI_ and temporal superior S_TS_ parts of the cornea indicated differences depending on pupil size (*p*-value respectively 0.028 and 0.013—last column in Table 1). The difference between 3 pupil sizes of S_TS_ is at the borderline of statistical significance (*p*-value = 0.05). On the other hand, S_NS_ do not depend on pupil size. Three of the four values of the angles did not show statistically significant differences when comparing the three sizes of the pupil. Only the superior corneal angle α_S_ was statistically different (*p*-value 0.017).

Taking into account pupil size and the geometrical parameters of the first order isochrome (sides lengths and interior angles values), statistically significant results were found mainly between the constricted and dilated pupils in case of the lengths of the sides (Wilcoxon test, S_TS_ and S_TI_, *p*-value = 0.01; S_NI_, *p*-value = 0.003) (Table 2). Significant differences were also found between the natural-sized pupil and the constricted pupil in the case of S_NI_ (*p*-value = 0.034). For the angle values, significant differences were found between the constricted and natural-sized pupil only for α_T_ (*p*-value = 0.041) and between the natural-sized and dilated pupil for α_S_ and α_I_ (*p*-value = 0.034 and *p*-value = 0.023, sequentially).

**Table 2:** Wilcoxon test for sides lengths (S_TS_, S_TI_, S_NS_, S_NI_) and angles sizes (α_T_, α_N_, α_S_, α_I_) in considered pupil sizes.

	S_TS_		S_TI_		S_NS_		S_NI_	
	N	D	N	D	N	D	N	D
C	0.050	**0.010**	0.158	**0.010**	0.480	0.050	**0.034**	**0.003**
N	–	0.158	–	0.050	–	0.388	–	0.308

	α_T_		α_N_		α_S_		α_I_	
C	**0.041**	0.158	0.099	0.754	0.099	0.239	0.480	0.158
N	–	0.814	–	0.099	–	**0.034**	–	**0.023**

*C* constricted pupil, *N* natural pupil size, *D* dilated pupil,

Bold denotes statistical significance.

The sides lengths and angle sizes of the first order isochrome were also compared separately for each pupil size (natural-sized, dilated, and constricted pupil) using the Wilcoxon test. Five of the six pairs of sides in each pupil size differed statistically significantly (S_TS_ vs. S_TI_, S_TS_ vs. S_NS_, S_TI_ vs. S_NS_, S_NS_ vs. S_NI_, *p-*value < 0.03 and S_TS_ vs. S_NI_, *p*-value < 0.05). The only side lengths that do not differ statistically are S_SI_ and S_NI_. Four of the six pairs of angle sizes differ significantly with each pupil size (α_T_ vs. α_N_, α_N_ vs. α_I_, α_S_ vs. α_I_, *p*-value < 0.03; α_T_ vs. α_S_, *p*-value < 0.05). Comparing the values of the angles in the nasal and superior cornea (α_N_ vs. α_S_), a significant difference was found in dilated and constricted pupils (*p*-value < 0.05). There was no significant difference only between α_T_ and α_I_ in each pupil size (*p*-value > 0.75). The statistical comparison (Wilcoxon test) was carried out on the geometrical parameters of the first order isochrome between the right and left eyes. There were no significant changes between the lengths of the sides and the values of the interior angles values (*p*-value ≥ 0.05) except for the α_S_ for the constricted pupil.

### Optical analysis

The determination coefficients *R*^2^ were designated from the linear approximation of the azimuthal distributions for three different pupil sizes. For the constricted pupil, the coefficients range from 0.977 to 0.995 (*p*-value < 0.001), for normal pupil size from 0.934 to 0.992 (*p*-value < 0.001), and for the dilated pupil coefficients range from 0.922 to 0.991 (*p*-value < 0.001). Student’s T-test revealed no statistically significant difference between R^2^ for three pupil sizes (*p*-value > 0.05).

Table 3 shows a comparison of the retardation and birefringence distributions of the horizontal section in the nasal and temporal corneas using the statistical method (analysis of the similarity between functions) described by Srihera et al.^40^. Statistical analysis revealed that for each pupil size the retardation and birefringence distributions between the nasal and temporal cornea are significantly different (*p*-value < 0.002). The differences were noticed in each measured eyes.

**Table 3:** Comparison of retardation distributions or birefringence distributions of nasal and temporal part of the cornea (*p*-value, Srihera method).

	*p*-value	
	Retardation	Birefringence
Nasal versus temporal		
Constricted	** ≤ 0.001**	** < 0.002**
Normal	** < 0.001**	** < 0.001**
Dilated	** < 0.001**	** < 0.001**

Bold denotes statistical significance.

**Figure 3:**
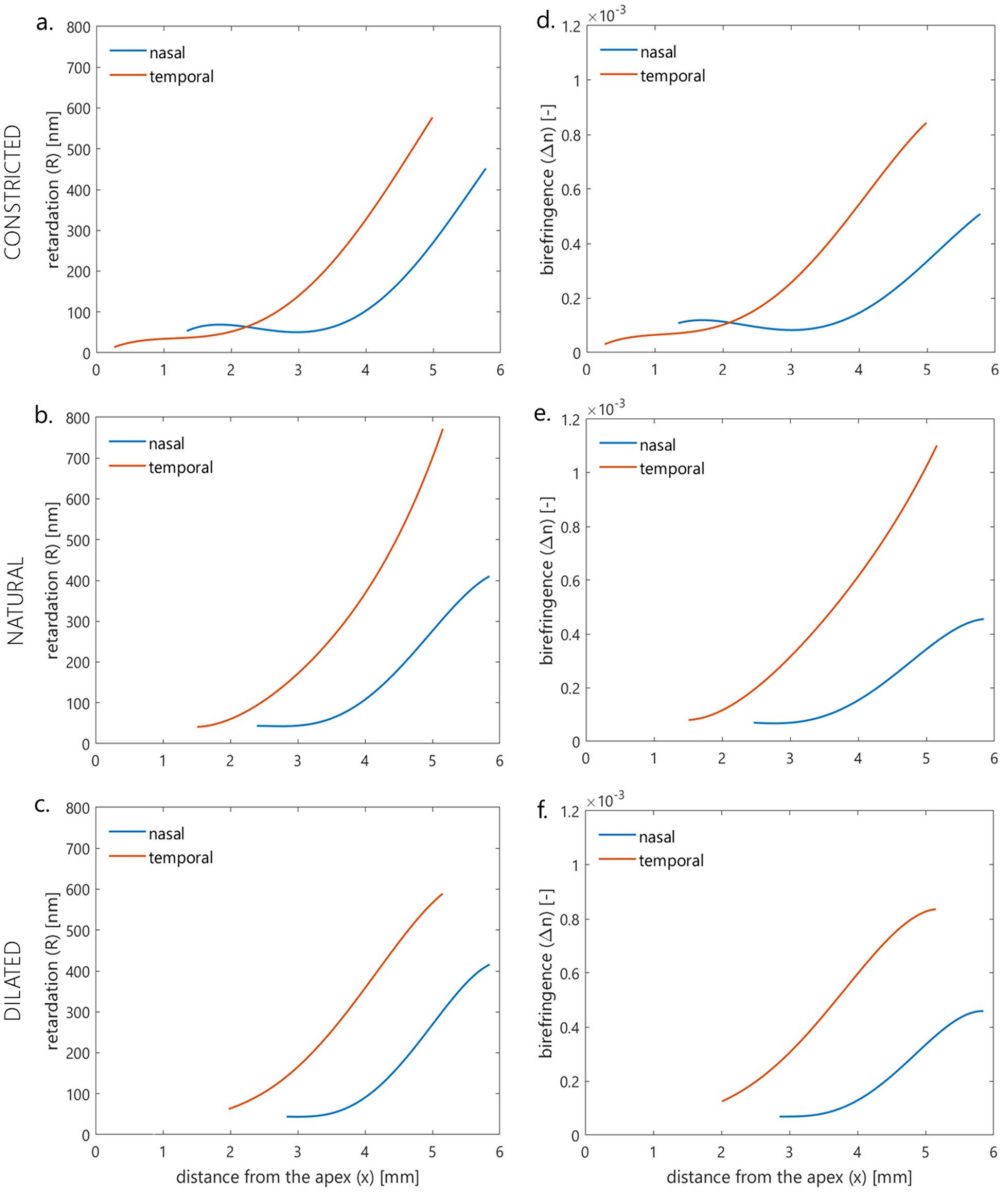
Juxtapositions of retardation (**a**,**b**,**c**) and birefringence (**d**,**e**,**f**) distributions in corneal nasal and temporal part for three pupil sizes—constricted (**a**,**d**), natural (**b**,**e**), and dilated (**c**,**f**).

To demonstrate the trends of the retardation and birefringence in nasal and temporal parts of the cornea the distributions were described by fourth-order polynomials (Fig. 3).

Table 4 includes the distributions comparison of retardation and birefringence separately for the temporal and nasal parts of corneas between different pupil sizes based on the Srihera’s statistical method. It was shown that the retardation distribution in the temporal part of the cornea is significantly dependent on the pupil size (constricted vs. dilated *p*-value < 0.002, constricted vs. natural, and normal vs. dilated *p*-value < 0.001; Table 4, column 3, line 3–5). The retardation distributions in the nasal cornea showed statistically significant difference between the natural-sized and the constricted pupil, natural-sized and dilated pupil (*p*-value < 0.001; Table 4, column 3, rows 7,8). In the case of the birefringence distributions, significant differences were noted between all three pupil sizes in both the temporal part (*p*-value < 0.002; Table 4, column 4, lines 3–5) and the nasal part of the cornea (*p*-value < 0.002; Table 4, column 4, rows 6–8). The same dependencies occur in every eye.

**Table 4:** Comparison of retardation distributions or birefringence distributions between different pupil sizes of nasal and temporal part of the cornea (*p*-value, Srihera statistical method).

	*p*-value	
	Retardation	Birefringence
Temporal		
Constricted versus dilated	** < 0.002**	** < 0.002**
Constricted versus normal	** < 0.001**	** < 0.001**
Normal versus dilated	** < 0.001**	** < 0.001**
Nasal		
Constricted versus dilated	> 0.05	** < 0.002**
Constricted versus normal	** < 0.001**	** < 0.001**
Normal versus dilated	** < 0.001**	** < 0.001**

Bold denotes statistical significance.

**Figure 4:**
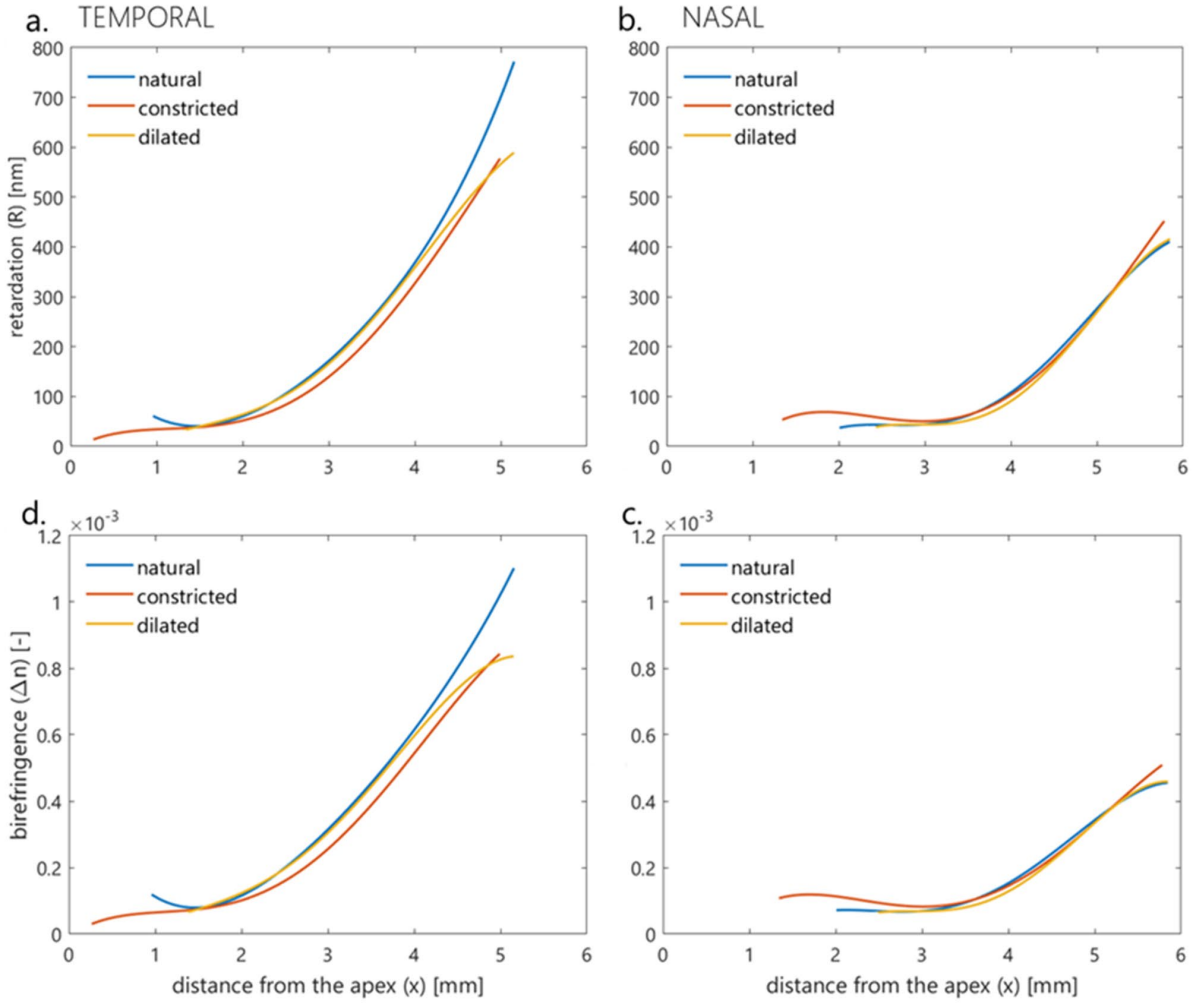
Juxtaposition of the retardation (**a**,**b**) and birefringence (**c**,**d**) distributions in three different pupil sizes of nasal (**b**,**c**) and temporal (**a**,**d**) part of the cornea.

To show the trends of changes of retardation and birefringence in nasal and temporal corneal parts in different pupil sizes, the distributions were described by fourth order of the polynomials (Fig. 4).

## Discussion and conclusion

The arrangement of the lamellae in the corneal stroma and their mutual orientations determine the anisotropic parameters of the cornea^19,29,41^, and alterations in their composition are a symptom of many corneal pathologies. Monitoring changes in the distribution of retardation (directly identified with birefringence) provides information about structural changes in the arrangement of collagen fibers, which may indicate ophthalmological diseases and some systemic diseases, e.g. keratoconus or diabetes mellitus^42–45^. Current optical methods that allow these diagnoses are rather complex, for example, PS-OCT, which allows imaging of the structure of the cornea at the microscopic level^42–44^ or confocal microscopy, but this method allows the examination of very small areas of the cornea at once. Corneal dystrophies (keratoconus, Fuchs dystrophy, Cogan dystrophy) require early diagnosis because changes in the corneal structure are irreversible. Keratoconus is a common corneal disease that is detected in most cases due to worsening visual acuity and untreated lead to the need for corneal transplantation. The exact cause of this disorder is still unknown. Gordon-Shaag et al. stated that environmental and genetic factors may contribute to its pathogenesis^46^ Diabetic keratopathy is the major complication correlated directly with diabetes mellitus. It is characterized by delayed corneal wound healing, recurrent corneal ulcers, and decreasing corneal epithelial sensitivity. The course of the disease includes, among others, changes in stromal structure or the loss of nerve fibers of the subepithelial plexus^47,48^. In diabetic stroma, one can find abnormal collagen fibrils bundles with altered thickness and spacing and increased thickness and tortuosity of stromal nerves. Early detection of such changes can help delay the development of pathologies before they affect visual acuity and quality of life. Quantitative changes in birefringence could indicate the appearance (early diagnosis) and subsequent development of the disease and progressive corneal pathology. Ophthalmological diagnostics supported by quantitative analysis of the birefringent properties of the cornea seems to be the future, but it is still a challenge for scientists. The availability of fast and non-invasive diagnostic techniques is still a problem. In this study, we used the partial Mueller matrix double-pass polarimeter as a non-contact measurement setup (described in detail in Sobczak et al.^39^) to measure the birefringent properties of the cornea. We took preliminary measurements for three pupil sizes: natural, constricted, and dilated. The obtained results were analyzed using geometric and optical methods.

First order isochromes are described by quadrangles and their geometrical parameters: sides lengths (S_TU_, S_TD_, S_NU_, S_ND_) and interior angles (α_T_, α_N_, α_U_, α_D_). Isochromes can be described using more complicated, closed figures, e.g. octagons. These introduce more parameters defining the nature of birefringence and greatly complicate the analysis. In our opinion, supported by previous publications^49^, using a quadrilateral is sufficient to determine the symmetry/asymmetry of the birefringence distribution in the cornea (especially the angle relations in the quadrilateral).

Isochromes (quadrilaterals) are not symmetrical^49,50^. This asymmetry may be caused by unequal extraocular muscles’ forces or/and the difference in lamellar distribution in the stroma. The locations of the inflection points of the quadrangles overlap with the attachments of the rectus extraocular muscles. The superior rectus and the inferior rectus muscles move the eye with almost the same force. However, there is a disproportion between the forces of the medial rectus and lateral rectus muscles^22^. In addition, there are differences in the structure of the cornea. Boote et al. showed that the density of the collagen fibrils changes within the cornea, and it is significantly higher in the sectors which correspond to the cardinal points^11^. They suggested that these extra collagen fibrils are supposed to maintain the shape of the cornea despite the influence of the extraocular muscle forces. The difference between values of interior angles in the nasal and temporal part of the cornea may result from uneven action of the horizontal rectus muscles. Levy et al. measured the central surface of the cornea using a scanning laser polarimeter with a variable compensator^51^. They showed a difference in retardation between the right and left eye with the natural size of the pupil. There are no reports in the literature on the impact of the size of the pupil on the recorded optical parameters of the cornea and whether this effect is the same for the left and right eye. In this work, we examined such a relationship and found that it is the same in the left and right eye for all three pupil sizes.

The sides of the quadrilaterals for constricted pupils were more elongated than for dilated ones, while the angles did not alter. However, statistical analysis revealed that the lengths of two of the four sides (S_TS_, S_NI_) and one quadrangle’s angle (α_S_) differ significantly when pupil size changes (Table 1). Comparative analysis for each geometrical parameter between pupil sizes showed that only for dilated and constricted pupils, three of the four sides lengths (except S_NS_) were significantly different (Table 2). For the remaining, statistical differences were not notable. Within one pupil size, the statistics showed that five of the six pairs of lengths of sides and four of the six pairs of interior angles values statistically differed. The geometrical approach to the analysis of the retardation results showed differences in the lengths of the sides and the internal angles values, but the differences are not statistically denoting. These differences may be due to changes in the geometry of the iris, which itself is not a symmetrical object, in terms of thickness, width, and volume^52^. Due to this asymmetry, asymmetric changes in iris geometry can be expected with pupil dilation/contraction. To our knowledge, the effect of changes in pupil size on changes in the structure of the iris, and thus on light scattering by the iris tissue, is not fully described in literature. Furthermore, changes in the iridial structure and the tension of the ciliary muscles may affect the corneal curvature in the peripheral area. This may affect the measurements, thus the shape of the isochromes^53,54^. Pharmacological dilation and constriction of the pupil impair the functioning of these muscles. The ciliary muscles play a crucial role in the accommodation process. Changes in the geometry of the eyeball towards a more oval shape, induced by contraction of the ciliary muscle, cause the peripheral corneal area to be steeper^55^. An alternative explanation can be given: the aqueous pressure may increase due to the change in shape and forward lens movement, which may act on the corneal surface and flatten the central area of the cornea^56^. The movement of the lens also causes the iris to shift and distort as its anterior surface changes its curvature.

The optical analysis consisted of comparing the cross-sections of computed azimuth angle and birefringence distributions. A high linear correlation of azimuth angle *α* distributions was observed for all pupil sizes. It could be stated that regardless of pupil size, the human cornea is medium with the radial distribution of the azimuth angle. Statistical analysis was performed for the horizontal distributions of retardation and birefringence, dividing them into two parts: nasal and temporal. Regardless of pupil size, values of the above parameters increase towards the limbus. For both retardation and birefringence, there were statistically significant differences between the nasal and temporal distributions for each of the three pupil sizes (Table 3). These results are consistent with previous results reported in literature^11,50^. Differences in corneal optical parameters (retardation, birefringence) between the temporal and nasal sections are probably caused by not only different orientations of the lamellae and their density but also the uneven increase of the corneal thickness towards the limbus. The graphical representation of the retardation (Fig. 3a–c) and birefringence (Fig. 3d–f) trends may show some similarities, especially in the middle parts of the distributions i.e. they are increasing functions with similar slopes. These results show that the observed retardation and birefringence (if they change) are due to changes in pupil diameter (which affects the shape of the iris) and not changes in the structural properties of the cornea. Statistically analyzing both distributions in the nasal and temporal parts of the cornea separately, but for three pupil sizes (constricted, natural, and dilated), there was no significant difference in the retardation distributions in the nasal part between constricted and dilated pupils. Other comparisons for retardation and birefringence distributions were statistically significant. The trends in the graphs show that statistically meaningful differences in distributions (both for retardation, Fig. 4a,b and birefringence, Fig. 4c,d) may be influenced by the marginal parts of the distributions that represent the peripheral (limbal) and the central area corresponding to the edge of the pupil. This may be due to changes in the shape of the pupil and the cornea in the limbal section. With a change in pupil diameter, the shape of the iris can change, thus changing the topography of the scattering surface as well as the shape and size of the diffraction edge. This leads to changes in measurement conditions, i.e. changes in corneal illumination with scattered light. Levy et al. showed that the retardation increases with the increase in pupil size, but their measurements concerned only the central part of the cornea^51^. They also revealed that cycloplegia or/and defocus decrease retardation and that pharmacologically pilocarpine-induced accommodation does not affect retardation. The change in corneal shape due to pupil size alteration may be the second reason for the disparity of retardation distribution, thereby birefringence distributions. As a result of the work of the iris muscles (the sphincter and the dilator of the pupil), the structure of the iris changes, which can cause changes in the anterior segment of the eye, i.e., in IOP. These changes may affect the peripheral geometry of the cornea^57,58^. It should be taken into account that the pupil was pharmacologically dilated/contracted, so this was an unnatural, excessive effect that may lead to changes in the limbal area of the cornea.

It is worth noting that most studies that measured birefringence of the cornea assessed the central corneal regions pertinent to vision^42–44,59–61^. Some of these studies were intended to aid ophthalmic diagnosis^42–44,59–61^. Using corneal birefringence characteristics in diagnosing disease requires sophisticated methods such as Polarization Sensitive Optical Coherence Tomography (PS-OCT), which can image corneal structure at the microscopic level. Studying the birefringence of the retinal fiber layer, Weinreb et al.^61^ used a polarization compensator to isolate the corneal birefringence. However, they did not specify the pupil diameter at which the measurements were made, and there is no information on whether it was controlled to be the same size.

This article presents the research method and results of the preliminary study. It focused on examining the effect of iris size/topography (altered by changing pupil diameter) on measured birefringent distributions in the peripheral cornea at the macroscopic level. To our knowledge, no one has studied such an effect, and it may have a significant impact on the measurements of the distribution of the azimuth angle and retardance as anisotropic parameters of the corneal structure.

These findings may be a factor supporting ophthalmological diagnostics because corneal birefringence is mainly due to the arrangement of collagen fibers in the stroma of the cornea. This is especially true for corneal ectasia, in which microstructural changes occur not only in the central part of the cornea, but also in the subsequent stages of its advancement, in its peripheral area, e.g. keratoconus. It seems essential to determine the optical parameters of the cornea with different pupil sizes in qualifying for refractive surgery. Any surgical procedure, such as cross-linking or refractive surgery, interferes with its structure and change corneal mechanical properties. This can affect the optical quality and, eventually, the procedure's success. The forces acting on the cornea after such operations will vary depending on the orientation of the fibrils that will manifest in the shape change of isochromatic fringes. More so, statistically significant differences were found in the lengths of the sides and the values of the internal angles of the quadrangle describing the first order isochromes between the three pupil sizes. All the more important is the repeatability and reliability of measurements before and after the procedure, and this may be influenced by pupil diameter. The presented results are interesting/attractive therefore the next step will be to extend the experiment to more healthy subjects as well as patients with corneal ectasias.

## Methods

The series of light intensity distribution maps were recorded using a partial Mueller matrix polarimeter described in detail in Sobczak et al.^38,39^. Briefly, the self-designed system comprises a polarization state generator (PSG, consisting of a linear polarizer, two liquid crystal variable retarders (LCVR)) placed directly in front of the subject’s eye, a light source (620 nm) with a collimating system, and a CMOS camera with an objective lens. The PSG works both as a set of polarizers and analyzers for six polarization states (four linear with azimuth angles equal to 0°, 90°, − 45°, + 45°, and two circulars). Due to the reflection from eye’s optical elements (mainly the iris), the system works as a crossed polarimeter. Acquired light intensity maps allow the designation of 7 elements of the Mueller matrix crucial for the computation of azimuth angle *α* and phase difference *γ* (retardation R) distributions. It needs to be noted that the light passes through the cornea twice, so the azimuth angle and phase difference values obtained from the computations need to be divided by 2. The corneal thickness distribution in horizontal cross-section was measured using the Scheimpflug camera in Corvis ST^®^ (OCULUS Optikgeräte GmbH; Wetzlar, Germany). This allows us to calculate birefringence, which in this article is defined as the ratio of retardation and corneal thickness.

The study included six healthy Caucasian volunteers (5 women and 1 man) in the age range of 26–59 years (average 37 ± 11.5) with no corneal surgery history. The spherical equivalents of refractive errors in the subject group were less than 2.00D. Study exclusion criteria included any systemic disease, history of ocular trauma or eye disease, refractive surgery less than 6 months before the study start date, conjunctival or intraocular inflammation, or corneal abnormalities. The study was approved by the Ethics Committee of Wroclaw Medical University (KB 329/2014) and adhered to the tenets of the Declaration of Helsinki. Informed consent was obtained from all participants after they had been fully informed of their requirements and about the purpose and procedures of the project.

The measurements were performed for three pupil sizes: (1) pharmacologically constricted (using Pilocarpinum WZF 2%, Polfa Warszawa), (2) natural-sized pupil, and (3) pharmacologically dilated (using Neosynephrin-POS 10%, Ursapharm) for both eyes. For each pupil size, the sets of light intensity maps were acquired sequentially three times for each eye separately. In the first part of the study, the measurements for natural-sized and dilated pupils were taken, and after 1 week, the study was completed with measurements for the constricted ones. All measurements were taken between 9 a.m. and 11 a.m. For each participant, firstly the right eye was measured then the left. During each measurement the subject had to look straight at the light focus point at a distance of 6 m and not to blink for 6 s. The measurements were taken in complete darkness.

**Figure 5:**
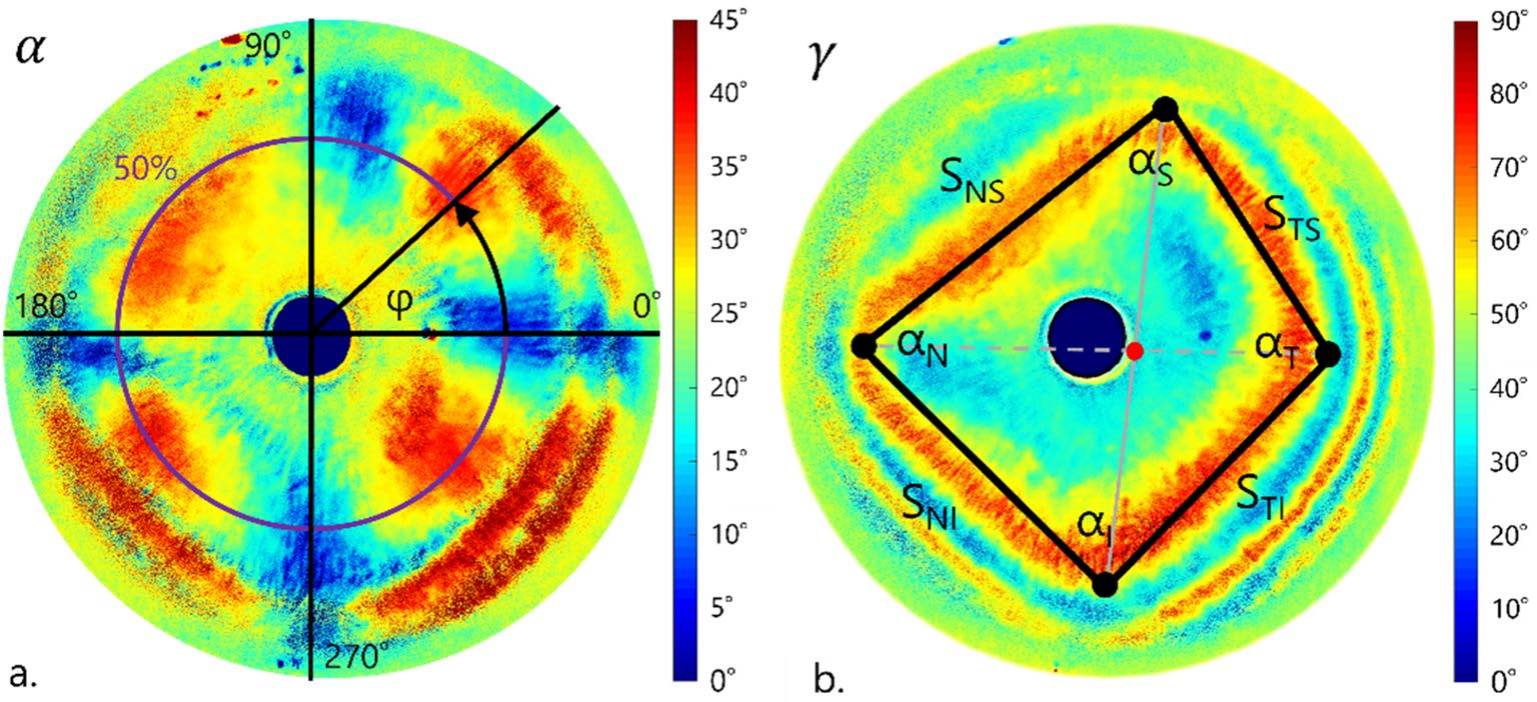
The azimuth angle *α* distributions with a scheme of transposition from cartesian to polar system (**a**), the retardation distributions described by a quadrilateral (sides lengths S_TS_, S_TI_, S_NS_, S_NI_ and interior angles α_T_, α_N_, α_S_, α_I_) (**b**).

From the azimuth angle α distribution maps, we designated the cross-section along half the distance from the corneal apex to the limbus (50% of half width of the image, purple line in Fig. 5a). The obtained data was presented in polar coordinates. Mueller matrix formalism imposes limitations on the azimuth angle *α* distributions results from 0° to 45° (Fig. 5a). Therefore, one should do the unwrapping procedure. Knowing that the azimuth angle *α* along the designated cross-section is an increasing function (explained in detail in Sobczak et al.^39^), when it reaches its first maximum, due to the unwrapping procedure, instead of decreasing, it continues to increase its values. The same happens in other function extremes. Received distributions were approximated by linear functions.

Maps of the phase difference *γ* distribution were analyzed geometrically and optically. The first order isochromes were described by quadrilaterals and their inflection points (black bullets in Fig. 5b). The n-th order isochrome is the line connecting the points where the optical path difference is the n-th multiple of the wavelength of the light source. From these coordinates were calculated the lengths of the sides (S_TS_, S_TI_, S_NS_, S_NI_) and angles values (α_T_, α_N_, α_S_, α_I_). The *γ* distribution maps were designated along nasal–temporal cross-sections (marked in Fig. 5b as a gray dashed line) and divided into two parts. The division point was a cross point of the diagonals of the quadrilateral (red dot in Fig. 5b). Due to the limitation of the used measurement method, the calculated values of the phase difference were in a range of [0°, 90°]. I makes the application of the unwrapping procedure necessary. With the use of thickness and retardation distributions, the birefringence distributions in a horizontal cross-section were calculated.

The repeatability measurements were conducted to confirm the reliability of the one exam. A tenfold measurement of each eye of one participant revealed that the standard deviation of computation of sides lengths and sizes of angles was lower than the reading accuracy. The maximum coefficient of variation was 1%. Analysis of the repeatability of azimuth angle *α* showed that coefficients of determination R^2^ for all distributions were higher than 0.970 (*p* < 0.001) hence the correlation was highly linear.

The statistical analysis of obtained parameters was performed using Statistica® (STATISTICA ver. 13.3, StatSoft Inc., USA). Sides lengths and angles values were checked for normal distribution using Shapiro–Wilk test. This hypothesis of normality however, was rejected. For these parameters, the non-parametric Wilcoxon test for dependent samples was used. Using the least square method the azimuthal distributions *α* were described and theirs approximation was specified by the determination coefficient *R*^2^. It was checked whether the distributions for three pupil sizes are statistically different using the T-test.

The distributions of the phase difference *γ* and birefringence may be described by two unknown regressions functions m_1_ and m_2_. To compare them, we used the method proposed in Srihera and Stute^40^. The approach is based on a comparison of the Nadaraya-Watson nonparametric estimators of m_1_ and m_2_. The test statistics $\tilde{T}$ incorporate properly standardized weighted differences of compared estimators computed at selected points. Under the null hypothesis H_0_: m_1_ = m_2_ (the equality of two regression functions that describes two compared distributions), the limit distribution of the test statistics $\tilde{T}$ is standard normal with mean 0 and variance 1. At the significance level $\beta$, the test rejects H_0_: m_1_ = m_2_ in favor of the alternative hypothesis H_1_: m_1_ ≠ m_2_ when $\left| {\tilde{T}} \right| > z_{1 - \beta /2}$. Here $z_{1 - \beta /2}$ is the $1 - \beta /2$ quantile of the standard normal distribution. Equivalently, H_0_: m_1_ = m_2_ is rejected, when the p-value corresponding to the observed value of $\left| {\tilde{T}} \right|$ is less than $\beta$.

## Data Availability

Data underlying the results presented in this paper are not publicly available at this time but may be obtained from the corresponding author—Marcelina Sobczak, upon reasonable request.
